# Childhood leukaemia, nuclear sites, and population mixing

**DOI:** 10.1038/sj.bjc.6605982

**Published:** 2010-11-09

**Authors:** L Kinlen

**Affiliations:** 1 Cancer Epidemiology Unit, University of Oxford, Richard Doll Building, Headington, Oxford, OX3 7LF, UK

**Keywords:** childhood leukaemia, leukaemia, acute lymphoblastic leukaemia, nuclear sites, population mixing

## Abstract

The excess of childhood leukaemia (CL) in Seascale, near the Sellafield nuclear reprocessing site in rural NW England, suggested that an epidemic of an underlying infection, to which CL is a rare response, is promoted by marked population mixing (PM) in rural areas, in which the prevalence of susceptibles is higher than average. This hypothesis has been confirmed by 12 studies in non-radiation situations. Of the five established CL excesses near nuclear sites, four are associated with significant PM; in the fifth, the Krummel power station in Germany, the subject has not been thoroughly investigated.

## Sellafield: an aetiological puzzle

Indications of an excess of childhood leukaemia (CL) near the Sellafield nuclear reprocessing site in Cumbria, north-west England, were announced in November 1983 in a TV programme. In the course of searching for adverse health effects in the workforce, the TV team discovered no less than 7 CL cases (mainly below age 10 years) over the previous 30 years in the coastal village of Seascale, 3 km from the site, together with two cases of the related malignancy non-Hodgkin's lymphoma (NHL), the overall expected number being less than one; the cases were attributed to radioactive discharges. Governmental response to the intense public concern was immediate: an advisory group was convened under Sir Douglas Black and produced its report within 7 months (Black, 1984). This confirmed the excess, but could not explain it in terms of radiation exposure. Other work was initiated, including a case–control investigation, and cohort studies covering all children born or attending school in Seascale. Shortly afterwards, the Committee on Medical Aspects of Radiation in the Environment (COMARE) was set up and further work was sponsored. Environmental discharges were found to be over 100 times too small to account for the excess, amounting to <10% of the total dose to Seascale residents (mainly from natural background), with a small chance of producing even a single case.

## Demography and epidemics

In 1986, an excess of CL was reported near Dounreay, Britain's other nuclear reprocessing site (Heasman *et al*, 1986). Here, the much lower radioactive discharges than at Sellafield prompted reflection on what other factor these two areas had in common. Seascale was a cul-de-sac in an isolated coastal area while Dounreay, on the northern edge of the Scottish mainland, was far from any conurbation; moreover, both had experienced marked population influxes: might this be relevant? Epidemics on islands have often been unusually severe, as in the case of measles in Faroe and of poliomyelitis in St Helena in the 19th century, and in Malta and Mauritius in the 20th century. As is now well understood, this reflects the importance for an epidemic of a high prevalence of susceptible individuals, which in isolated places is promoted by the reduced opportunities for contacts with a wider infective pool. Indeed, what often ends an epidemic is the reduction, caused by the outbreak itself, in the prevalence of susceptibles below some critical level. In addition to its relative isolation, Seascale also had by far the highest socioeconomic make-up of any rural parish in England, due to the nuclear body's allocation of houses to scientists and other senior Sellafield staff. This policy would further promote a high prevalence of susceptible individuals, as the associated high standards of hygiene can protect against infective exposures, particularly early in life. Thus, in polio outbreaks in pre-immunisation days, paralysis was more frequent among children of the better off, who lacked the immunity gained by other children from their frequent early-life exposures.

## Subclinical infection and CL

In fact, an infective origin in CL has been a long-standing suspicion, encouraged by reports of apparent ‘clusters’ (although here chance must often be involved), and by the discovery of the viruses underlying the leukaemias in several animal species and in human (HTLV-1). However, such is the emotive power of radiation that it had effectively pushed infection out of mind. As CL is not contagious (*marked* space–time clustering being absent), it could belong to that large category of illnesses, which are rare responses to some more common infection. Such infections, in which age at exposure and microbial dose are considered important determinants of whether *illness* will occur, are mainly subclinical, and their effects largely immunising. Subclinical infections were considered by Macfarlane Burnet to be central to any understanding of the epidemiology of infectious disease; they include poliomyelitis, infectious mononucleosis, and hepatitis A, besides the more than 10 cancers (e.g. of cervix and liver) that are already known to be caused by viruses (Kinlen, 2004). As the history of microbiology underlines the virtual synonymity of infectivity with the action of s*pecific* microbes, evidence of infectivity in acute lymphoblastic leukaemia (ALL), the dominant type of CL, is likely also to point to a specific (probably viral) agent.

## The hypothesis of rural population mixing

Wars, with their social disruption and population mixing (PM) have long been known to precipitate, both among servicemen and civilians, epidemics of meningococcal meningitis, otherwise a mainly sporadic illness – Britain experienced outbreaks in both world wars. Parents, teachers, and general practitioners see at first hand how schools act as mixing fields for the transmission of the common infections of childhood. Among cats, feline leukaemia virus (FeLV) infection is widespread, but in singly kept animals, cases of leukaemia are uncommon (i.e. ‘sporadic’ in type); in contrast, when they are subject to unusual mixing with consequent heavy exposures, as in so-called multi-cat households, incidence is greatly increased (i.e. ‘epidemic’ in type). Relating such observations to the two ‘nuclear’ excesses led to the hypothesis of rural PM. Its basic grounds are well established: that epidemics require sufficient susceptible individuals and that these are more prevalent in rural areas, in which the lower population density reduces contacts with a wide infective pool. The hypothesis holds that, while infection-based leukaemia is widespread throughout the childhood population, a localised *epidemic* of the underlying infection would be promoted by a large influx of mostly urban outsiders into a rural area, because of the consequent increased level of contacts between susceptible and infected individuals; and that in turn this would tend to produce an excess of the infection's uncommon complication, CL (Kinlen, 1988, 1995).

## Testing the PM hypothesis

The PM hypothesis has now been tested with respect to CL in almost all major examples of marked rural–urban mixing in Britain over the past 70 years. These include rural new towns (Kinlen *et al*, 1990), wartime evacuation of children to rural areas (Kinlen and John, 1994), post-war increases of national servicemen in rural areas (Kinlen and Hudson, 1991), rural Scottish communities from which many men worked away from home in the North Sea oil industry (Kinlen *et al*, 1993), Scottish hydro-electric schemes, areas around large rural (non-nuclear) construction sites (Kinlen *et al*, 1995), and wartime Orkney and Shetland in which large numbers of servicemen were stationed (Kinlen and Balkwill, 2001). In all these situations, significant, but transient excesses of CL were found and, in the last five of them, the incomers did not include children, indicating the importance of adults in transmitting the infection. In keeping with the hypothesis of an infective basis, risk was highest among children whose fathers had occupations involving many community contacts, including teachers (Kinlen, 1997). In contrast, no excesses were found in urban areas that were subject to similar degrees of PM, pointing to an immunity to epidemics produced by earlier widespread exposure to the relevant agent(s). At least five studies of rural PM outside Britain have also found excesses, in Ontario, Canada (Koushik *et al*, 2001), in the New Territories, Hong Kong (Alexander *et al*, 1997), near La Hague, France (Boutou *et al*, 2002), in Greece (Kinlen and Petridou, 1995), and in the United States (Wartenberg *et al*, 2004). Recently, a striking excess of CL occurred in the small desert town of Fallon, Nevada, United States as the annual numbers of trainee recruits passing through the nearby naval air base peaked at 50 000 in the year 2000, an extreme example of rural PM (Kinlen and Doll, 2004).

## CL and NHL (in Britain)

An independent review of the CL and NHL cases in the Thurso–Dounreay area revealed that two NHL cases were in fact leukaemias (COMARE, 1988). As such a review had not been performed for all Scotland, these two cases could only be included in an analysis of CL and NHL combined; only when this was performed, did the excess within 25 km (in 1968–1984) reach statistical significance (0–24 years: *P*=0.039). It was recommended that, as these related malignancies might be confused elsewhere, future studies of this type should examine them together, and this has as been the practice in Britain.

## Other hypotheses

Radioactive discharges. Although initially seen by some as an obvious explanation, radioactive discharges have not been implicated in any CL excess near a nuclear site (see also section ‘Sellafield: an aetiological puzzle’).
Pre-conceptional paternal irradiation (PPI). In the case–control study instigated by the Black Committee, a significant relation was found between CL (and CL-NHL) risk and the cumulative PPI dose; furthermore, this was claimed to explain the Seascale excess (Gardner *et al*, 1990). This study had been limited to children resident *and born* in west Cumbria, because the Seascale risks appeared to be restricted to children born in the village. However, the subsequent demonstration of a significant excess among Seascale children born outside the village, not attributable to PPI, indicated that this hypothesis could not explain the whole excess, and had been derived from a subgroup (Kinlen, 1993). No support for PPI as a cause of CL has emerged from studies of the offspring of atomic bomb survivors, or of nuclear workers elsewhere in the world (COMARE, 2002); nor did it contribute to the excess recorded near Dounreay, though there was no scarcity of men there with high PPI doses.
The Greaves’ delayed infection hypothesis. This proposed that ALL is caused by antigenically produced mutations in lymphocytes that had escaped the postulated differentiating effects of (non-specific) infections in the first 2 years of life, which would normally be protective (Greaves, 1988). However, this will not explain the PM-associated excesses, which have not spared the youngest ages; indeed, they were maximal at these ages in one study (Kinlen and Hudson, 1991). More recently, the large UK Childhood Cancer Study, which was partly designed to test the Greaves’ hypothesis, found that infections in the first year of life were not protective, but were associated with a significant CL excess (Roman *et al*, 2007).

## CL and PM near reprocessing sites

The significant positive findings summarised in section ‘Testing the PM hypothesis’ represent strong evidence for PM being responsible for the excesses near Sellafield and Dounreay: not only were they the source of the hypothesis under test, but the isolation and influxes there were extreme, the latter greatly increasing the 1951 populations of nearby Seascale and Thurso, respectively. Furthermore, subsequent to the discovery of the CL excess near Sellafield, when the play of chance had less scope, the excess became still more marked during the construction on that site of the massive Thermal Oxide Reprocessing Plant (THORP) in 1983–1993 by around 50 000 workers – the largest construction project and perhaps the most extreme example of rural PM in the United Kingdom. The associated excess of CL-NHL is the highest recorded in the United Kingdom (15-fold at ages 0–24 years; Table 1).
The older age at CL diagnosis among incomers. In both the above ‘nuclear’ excesses, in contrast to cases at ages 0–4 years, most of those diagnosed at older ages were in incomers (Table 2). This pattern recalls classic observations in Burkitt's lymphoma, a mainly childhood malignancy in the lowland Ugandan endemic area, whereas cases at older ages were almost invariably among incomers from low-incidence areas; this pattern was interpreted as reflecting the high level of immunity among the older locally born residents, due to their early exposure to widespread subclinical EBV infection (Burkitt and Wright, 1966). (The relative immunity of urban areas mentioned earlier refers to *epidemics*: sporadic CL still occurs there, and urban children coming into an existing epidemic/excess would be at risk, as in Seascale). Another feature of the ‘nuclear’ excesses is that the older CL cases (ages 5–24 years) tended to occur later in the course of the epidemic than those at 0–4 years (Table 2), a pattern noted in feline leukaemia. In contrast to kittens, protracted exposure appears necessary among older animals before overt FeLV infection develops (Grant *et al*, 1980).
La Hague. The only other large nuclear reprocessing plant in Europe is La Hague in France. No excess was found in its surrounding area overall (Guizard *et al*, 2001); however, this includes urban areas and it is notable that a significant increase of CL was recorded in those local *rural* areas, which were markedly affected by construction-related PM (Boutou *et al*, 2002).

## PM near, but unrelated to, a nuclear site

The presence of a nuclear site near CL cases tends to distract consideration of other possible aetiological factors – such as PM. The North Sea oil industry has involved PM of extreme degree. Certain aspects of the huge undertaking to bring ashore Britain's North Sea oil were especially conducive to intense mixing of men from the most remote and the most urban parts of Britain, many thousands of workers being brought to Shetland and Orkney to build the large terminals there, north of the Scottish mainland. Contacts with children did not take place near the worksites, as few lived nearby, so the focus of a study was on workers’ home areas, to which they returned each month. After workforce numbers reached unusually high levels, significant increases in CL occurred in 1979–1983 in those rural postcode areas that had many oil worker residents. What was unexpected was finding that the Thurso–Dounreay area was high in its prevalence of oil workers: the excess there was related not to nuclear work, but to the oil industry (Kinlen *et al*, 1993). This also explained why the main CL increase near Dounreay began >20 years after the plant was built. This does not mean that the initial influx into the area to build and operate the nuclear facility had no effect on CL: in the period 1951–1967 when Thurso grew markedly, a two-fold increase occurred, though the small numbers did not reach statistical significance (Kinlen, 1988).

Another example of PM near a nuclear site causing confusion has occurred near the Royal Ordnance Factory at Burghfield in Berkshire, England, a site with trivial radioactive releases. Here, a significant CL excess, mainly at 0–4 years within 10 km (but not within 5 km), in 1972–1985 reflected the inclusion of Reading (Roman *et al*, 1987). For the same period, a study of commuting, a distinctive and prevalent type of PM in modern life, found this county district to rank highest for increases in census-based measures of commuting and also of population growth. These showed a significant relation with CL, both in the trend across all districts covered and in the five county districts in the highest decile of commuting increase (Kinlen *et al*, 1991). This study, though repeatedly overlooked (COMARE, 1996, 2005, 2006), also indicates that PM-related excesses may occur in urban areas, at least when surrounded by extensive rural areas.

## Other evidence for infection underlying CL

Rural PM studies are not the only source of evidence of an infective origin in CL. The slight (but statistically significant) space–time clustering in the absence of marked rural PM is most easily explained on infective grounds. Like certain childhood infections, the higher incidence reported among first-born children, as in a recent national study (Dockerty *et al*, 2001), is consistent with them bringing immunising doses home to their younger siblings. Such exposures are implied by the lower CL incidence after early child care as found in many studies (Urayama *et al*, 2010). The contrasting effects of PM in rural and urban areas parallels the striking difference noted when the United States mobilised an army in World War I: camps with men drawn from the sparsely settled states had a far higher incidence of infections than camps (sometimes close by) with city-bred recruits (Love and Davenport, 1919). Also, in case–control studies, CL risk showed a significant positive trend across increasing levels of paternal occupational contacts (Kinlen and Bramald, 2001; Kinlen *et al*, 2002), consistent with the observations in cytomegalovirus infection and in some poliomyelitis epidemics; again, the role of adults in transmission is highlighted. These paternal effects were seen in rural, but not in urban, areas.

## Implication of the PM findings

The hypothesis focuses on rural PM, not because it was envisaged as the main way in which either infection is transmitted or CL occurs, for this would be nonsense, but because, though rare, it represented a situation conducive to an epidemic or mini-epidemic that was *testable*. If CL is predominantly infective in origin, a variety of situations must on occasion (as in any infection) be conducive to a localised outbreak of the infection as a result of the myriad interplay of contact patterns, dose and distribution of susceptibles. Consistent with this is the widespread, apparently non-random variation recently found in a detailed study of CL in Britain in 1969–1993 down to the electoral ward level (COMARE, 2006), the slight (but significant) space–time clustering, and also the increased incidence reported in rural areas of high social class (Alexander *et al*, 1990) and in some places in which nuclear sites were planned, but not built (Bithell *et al*, 1994).

## Objections to the PM hypothesis in relation to nuclear sites

The size of the Seascale excess. COMARE in its Fourth Report (1996) rejected PM as an adequate explanation for this excess on the grounds that it could not explain either its magnitude or its duration. However, the former involved the pitfall pointed out before the report, of not comparing like with like (Kinlen *et al*, 1995). For the period 1953–1983, the Committee focussed on Seascale, the parish with the highest CL incidence in the area. In contrast, PM studies even handedly covered large areas that met pre-defined criteria, comprising many, in some cases thousands of, parishes although among these, were some with large excesses (see Table 1). The correct comparison is with an area *around* Sellafield, as it was this plant that first drew the TV team to the area. When the area within 10 km is examined, as in a study of large rural (non-nuclear) construction projects, the magnitudes of the excesses are closer (Table 1; Kinlen *et al*, 1995). It is, of course, legitimate to focus on Seascale in the period *after* the TV programme, as an hypothesis had then been generated: 3 cases below age 25 occurred (expected 0.19; O/E 15.8) in the years 1984–1993. Although COMARE correctly stressed this excess, no mention was made of the construction of THORP, or of its particular relevance (because of its scale) to the PM hypothesis. Their observation that PM had not accounted quantitatively for the Seascale excess was addressed by Dickinson and Parker (1999), who applied a model of PM to CL-NHL data for children born in Cumbrian wards other than Seascale: the largest excess was predicted in Seascale and, allowing for the play of chance, its size was close to what was observed.
The duration of the excess. In regarding the prolonged duration of the Seascale excess as a problem for the PM hypothesis, COMARE overlooked another aspect, which had been stressed – that a high level of susceptibles would be maintained by the exceptionally high turnover of its child population: 43% of all children born there moved away before age 5, while half of those who were not born there, but attended its primary school, had moved on again before age 11, with a corresponding inward flow (Gardner *et al*, 1987). Infection would also be promoted by the almost continuous presence of construction workers on the Sellafield site. The occurrence of a significant CL cluster in nearby Egremont North (Craft *et al*, 1993) is notable as it was the local centre for the building contractors’ migrant workforce (Kinlen, 1995). The official Scottish evaluation of the CL-NHL excess near Dounreay (Black *et al*, 1994) made no mention of its starting at the same time as in other areas affected by the oil industry, but instead stressed that it persisted longer, implying that this was a problem for the hypothesis. As in the Seascale excess, cases occurring later in the epidemic mainly involved older incomer children, of which the area had an unusually high prevalence (Kinlen *et al*, 1993; Table 2). Inevitably, areas having many oil worker residents, but few incomer children cannot show such an effect.
An unconfirmed prediction. COMARE (1996) claimed that the PM hypothesis predicted a CL excess in Seascale during the Second World War, when ordnance factories at nearby Sellafield and Drigg were constructed and operated by large workforces; and that many houses were built in Seascale for these workers. No excess, they stressed, had occurred. In fact, the houses in question were built *after* the war: the date of the relevant document had been misread. This objection also overlooks the small size of the village in those years, when the annual school roll showed only 41 children aged 5–15 years, and even a single CL case would have represented an 80-fold increase. More recently, a wartime excess has been demonstrated – not in Seascale, but in other areas of west Cumbria where most ordnance factory workers lived (Kinlen, 2006).
No agent identified. It was the epidemiological evidence of infectivity that led to the discovery of the viruses underlying Burkitt's lymphoma, cervical cancer, and Kaposi's sarcoma, as well as the microbial agents responsible for a host of other infective disorders. Certain statements about the lack of an identified agent in CL (Laurier *et al*, 2008a) can suggest that (even strenuous) efforts have failed to find a previously unidentified virus with the characteristics to which rural PM studies point. However, this is not the case. An investigation by MacKenzie *et al* (2006) involved blood from *urban* cases, their controls consisting of the same children in remission or their parents. With notable exceptions (Doll, 1999), it would seem that, without prompt discovery of the relevant agent, evidence of infectivity is for many unpersuasive. Whether the relative virological neglect of CL reflects the demands of HIV research, or the (probably) mistaken belief that only specimens from a PM-related excess are relevant, is not clear.

## CL and nuclear sites in different countries

Table 3A lists the main geographical studies of CL incidence within specified distances of all nuclear facilities in a given country (based on Laurier *et al* (2008a)). (Mortality studies were excluded because of the possibly variable effects on rural fatality rates of early treatments.) In no country was a significant overall excess evident in any proximity category although, in western Germany, there was weak evidence of an excess at 0–4 years within 5 km of its 16 nuclear power stations (O/E 1.41; 95% CI: 0.98, 1.97; Kaatsch *et al*, 2008a); no significant excess in this age group and proximity zone has been found in France (Laurier *et al*, 2008b) or Britain (Bithell *et al*, 2010). Within the multiple-site studies listed, >60 sites were also examined individually, but no significant excess was detected. However, in the case of four British sites: Sellafield, Dounreay, Burghfield, and the Rosyth naval dockyard, statistical tests such as the linear risk score, incorporating a distance-from-the-site measure (Bithell *et al*, 1994), gave significantly raised values (COMARE, 2005; Table 3A), although in the case of Rosyth, the distance effect was no longer significant when one additional year of observation was included (Sharp *et al*, 1996). These effects are consistent with the greater PM in Seascale, west Thurso, and Reading than in the more peripheral parts of these circles.

Studies restricted to the Sellafield, Dounreay, and Burghfield areas have also found a significantly increased incidence of CL and NHL (Table 3B), and the negative findings for these sites in Table 3A reflect the narrower coverage of ages and time periods. Notably, in the vicinity of all three sites, nationally extreme levels of PM have been recorded (see sections ‘Demography and epidemics’, ‘CL and PM near reprocessing sites’, and ‘PM near, but unrelated to, a nuclear site’). Near a fourth site (La Hague, France) a significant CL excess has occurred in nearby rural (but not urban) areas with a high level of construction-related PM (Boutou *et al*, 2002). An increased incidence of CL within 5 km of the Krummel power station in western Germany, most marked at ages 0–4 years, has occurred over the period 1990–2005, first detected in 1992 (Hoffmann *et al*, 2007), raising the question whether Krummel is also associated with PM. This excess is responsible for the weak evidence mentioned above of increased CL incidence at 0–4 years within 5 km of all nuclear sites in western Germany; by excluding Krummel, this is removed (O/E ratio 1.20; CI: 0.78–1.76; Table 4).

A different approach to these German cases at ages 0–4 years has been used in the ‘KiKK’ case–control study, namely a comparison of the distances from the plants of affected children's homes with the corresponding distances for those controls who could be included, in which the former were closer than the latter (Kaatsch *et al*, 2008a, 2008b; Darby and Read, 2009). These findings, interpreted as indicating an increased risk near the plants, should be reflected in a corresponding marked increase in CL incidence, and yet this has not been found (Table 4). As incidence details have not been presented for sites other than Krummel, certain assumptions had to be made in deriving their expected values in Table 4. Here, the absence of a significant excess was robust to doubling the (assumed) expected values for Krummel in the earlier operating years of 1984–1989 (thereby increasing the O/E ratio for the other sites: 1.24; 95% CI: 0.81, 1.82). No reasons have been given for questioning the reliability of either the German population estimates (or the 1987 census counts) for the relevant communities, or the calculated distances of community centroids to specific points (i.e. power stations), which together represented the basis for the estimation of incidence both around Krummel as well as the other sites. The similar CL numbers given by the two approaches, whether living within 5 km of the plants (37, as determined by individual measurement) or belonging to communities with centroids in this zone (34), hardly support the view that the latter are ‘much less exact’ (Kaatsch *et al*, 2008a). Rather, they point to a problem with the KiKK study controls and the complexity of their selection process; a detailed comparison of the two data sets by site and time period might locate this more specifically. Importantly, incidence cannot be deduced from a case–control study, and its results cannot obviate population-based incidence findings.

PM has been discounted near Krummel because, ‘although some in-migration and PM may have occurred’, its population size was ‘fairly stable over the previous two decades’ (Hoffmann *et al*, 2007). Similar comments could also be made, however, about certain PM-associated excesses of CL, in which the PM either occurred between censuses, or away from home, or involved mobile construction workers, whose regular turnover on worksites is not reflected in the numbers present at any given time. Aspects of the Krummel excess recall demographic features mentioned earlier in this review, including its being most marked in rural areas separated from the plant by the River Elbe in which crossing places are limited. However, the occupational characteristics (including temporary foreign workers) and movement patterns in this area have not received attention comparable with that near British and French reprocessing sites.

## Conclusion

An infective basis in CL, albeit not specifically identified, is strongly indicated by the significant relation of CL excesses with marked rural PM found in some 12 studies in 6 countries. It is striking that this same relation has also been found around, or near, each of the three large European nuclear reprocessing sites, of Sellafield, Dounreay, and La Hague, and also near the ordnance factory of Burghfield, though here as well as near Dounreay, the PM was unrelated to the nuclear site. Nuclear power stations, being smaller operations than reprocessing sites have not usually been associated with marked PM and, in several countries, they have shown no excess incidence in any age group or proximity category. The area within 5 km of the Krummel plant in Germany represents an exception, but here, PM (and related factors), whether or not connected with the plant, have not been thoroughly investigated.

## Figures and Tables

**Table 1:** Leukaemia and NHL at 0–14 years within 10 km of large construction projects and Sellafield (and in the parish having the largest excess)

**Observed (Obs) to expected ratios (O/E) (observed numbers)**		
	**Within 10 km of**	**Parish with largest excess**
	**O/E (Obs)**	**O/E (Obs)**
*British construction*		
Projects 1951–1993^a^	1.9 (59)^**^	Nr. Drax^b^ 8.0 (5)^**^
		
Sellafield^c^ 1951–1983	2.5 (12)^d^^,^^*^	Seascale 11.0 (8)^***^
		
Sellafield 1984–1993^e^	3.2 (4)	Seascale 8.3 (1)^f^

Abbreviations: NHL=non-Hodgkin's lymphoma; THORP=Thermal Oxide Reprocessing Plant.

^*^P<0.05; ^**^P<0.01; ^***^P<0.001.

a Non-nuclear power stations, oil refineries, etc. (Kinlen *et al*, 1995).

b Near Drax, largest coal-fired power station in Europe.

c Sellafield, largest nuclear site in United Kingdom.

d Leukaemia only before 1968.

e THORP construction period (post-TV).

f O/E 1984–1993 at ages 0–24 years: 15.8^**^ (3).

**Table 2:** Leukaemia and NHL in young people in Seascale and the Thurso–Dounreay area (from Kinlen, 1993)

**(A) Median calendar year of diagnosis by age (number of cases)**		
**Area**	**0–4 years**	**5–24 years**
Seascale	1968 (5)	1981 (6)
Thurso–Dounreay	1980/1981 (4)	1984 (7)
		
**(B) Observed to expected ratios at 5–24 years by birth place (number of cases)^a^**		
**Area**	**Born within**	**Born outside**
Seascale	3.6 (1)	13.0 (5)
Thurso–Dounreay	1.2 (2)	3.0 (5)

Abbreviation: NHL=non-Hodgkin's lymphoma.

a All 5 aged 0–4 in Seascale, and all 4 aged 0–4 in the Thurso–Dounreay area were also born within there.

**Table 3a:** Childhood leukaemia incidence around nuclear sites

**Multiple-site studies, by country (based on Laurier *et al*, 2008a, and updated^a^). Studies listed are not all independent**										
	**Sites**	**Radius**		**Age**	**Observed^b^**	**Expected^c^**		**Individual sites**		
**Country**	**No. type**	**(km)**	**Period**	**(years)**	**(O)**	**(E)**	**O/E**	**O/E**	**LRS/MLR *P*-value**	**Reference**
England and Wales^d^	23 All	25	1967–1987	0–14	3534	3580.8	NS	NS	S^***^, B^*^	Bithell *et al* (1994)
Scotland^d^	7 All	25	1968–1993	0–14	399	410.9	NS	NS	D^*^	Sharp *et al* (1996)
Britain^d^	13. PG	25	1969–1993	0–14	692	721.14	NS	NS	NS	COMARE (2005)
—d	15. Other	25	1969–1993	0–14	2318	2309.61	NS	NS	S^*^, D^*^, B^*^, R^*^	COMARE (2005)
Britain	13. PG	5	1969–1993	0–4	20	14.74	NS	NA	NA	Bithell *et al* (2010)
W. Germany^e^	20. All	5, 10, 15	1980–1990	0–14	30	27.2	NS^h^	NA	NA	Michaelis *et al* (1992)
—e	20. All	5, 10, 15	1980–1990	0–4	19	15.1	NS^h^	NA	NA	Michaelis *et al* (1992)
W. Germany^e^	20. All	15	1991–1995	0–14	182	178.4	NS	NA	NA	Kaatsch *et al* (1998)
—e	20. All	15	1980–1995	0–14	461	456.4	NS	NA	NA	Kaatsch *et al* (1998)
E. Germany	3. All	15	1979–1988	0–14	19	15.1	NS	NK	NA	—f
E. Germany^e^	3. All	15	1991–1995	0–14	19	21	NS	NK	NA	—f
W. Germany	15. PG	5, 10, 30^g^	1980–2003	0–4	34	24.09	See Table 4	NA	NA	Kaatsch *et al* (2008a)
France	19. PG	5, 10, 15, 20	1990–1998	0–14	8	10.64	NS^h^	NS	NS	White-Koning *et al* (2006)
	8. Other	5, 10, 15, 20	1990–1998	0–14	57	64.47	NS^h^	NS	NS	White-Koning *et al* (2006)
	20. ALL	5, 10, 15, 20	1990–1998	0–4	39	40.04	NS^h^	NA	NA	White-Koning *et al* (2006)
France	19. PG	5, 10, 15, 20	1990–1998	0–4	5	5.2	NS^h^	NA	NA	Laurier *et al* (2008a)
Canada (Ontario)	5. All	25	1964–1986	0–14	95	88.8	NS	NS	NA	McLaughlin *et al* (1993)
Finland	2. PG	15, 50	1975–2004	0–14	11	9.82	NS	NS	NA	Heinavaara *et al* (2010)

Abbreviations: S=Sellafield; D=Dounreay; B=Burghfield; R=Rosyth; NA=not applicable; LRS=linear risk score; MLR=maximum likelihood ratio; PG=power-generating site; NS=not (or none) significant; NHL=non-Hodgkin's lymphoma; CL=childhood leukaemia.

^*^P<0.05; ^***^P<0.001.

a Excluded: mortality studies, county-based incidence studies, those without observed and expected numbers, and those of potential nuclear sites.

b Numbers refer to the lowest radius zone.

c National rates (province-wide rates were used for Ontario); in contrast to the others, the west German registry has a single ascertainment source.

d CL+NHL.

e Acute leukaemia.

f Quoted in Laurier *et al* (2008a).

g And also 50 and 70 km.

h Nor with other radii.

**Table 3b:** Studies restricted to specific sites, by country

**Country**	**Site**	**Type**	**Radius (km)**	**Period**	**Age (years)**	**Observed**	**Expected^a^**	***P*-value**	**PM**	**Reference**
England	Sellafield^b^	RP	10	1951–1993	0–14	16	6.05	—^**^	+	Kinlen *et al* (1995) c
England	Burghfield^b^	OF	10	1972–1985	0–4	27	12.19	—^**^	+	Roman *et al* (1987)
			10	1972–1985	0–14	38	23.86	—^*^	+	Roman *et al* (1987)
			5	1972–1985	0–4	6	2.77	NS		Roman *et al* (1987)
			5	1972–1985	0–14	7	5.46	NS		Roman *et al* (1987)
Scotland	Dounreay^b^	RP	25	1968–1991	0–14	9	3.49	—^**^	+	Black *et al* (1994)
			25	1968–1991	0–24	12	5.17	—^**^	+	Black *et al* (1994)
France	La Hague	RP	10	1978–1998	0–24	5	2.30	NS^d^	+	Guizard *et al* (2001)
Western Germany	Krummel	PG	5	1990–2005	0–4	10	2.04	—^***^	NK	Hoffmann *et al* (2007)
			5	1990–2005	0–14	14	4.00	—^***^	NK	Hoffmann *et al* (2007)

Abbreviations: NK=not known; NS=not significant; RP=reprocessing site; OF=ordnance factory; PG=power-generating site; PM+=marked population mixing; NHL=non-Hodgkin's lymphoma; CL=childhood leukaemia.

^*^P⩽0.05; ^**^P⩽0.01; ^***^P<0.001.

a Based on national rates except for La Hague, for which regional rates were used.

b CL+NHL.

c Corrected (Table 1).

d At ages 0–4, 0–14, and 0–24 within 10, and up to 35 km, NS; (in rural areas <35 km with high PM, 10 cases aged 0–24, 4.77 expected ^*^ Boutou *et al*, 2002).

**Table 4:** Leukaemia at 0–4 years within 5 km of nuclear power stations in Western Germany

**Nuclear sites**	**Observed (O)**	**Expected (E)**	**O/E**	**95% confidence intervals (CIs)**	**Reference**
All sites (1980–2003)	34	24.09	1.41	0.98, 1.97	Kaatsch *et al* (2008a)
Krummel (KR) (1984–2003)	8	2.43†	3.29	1.41, 6.52	Hoffmann *et al* (2007)
All except Krummel^a^	26	21.66	1.20	0.78, 1.76	By subtraction^b^

a Expected value for 1984–1989 not given, but taken as half that for ages 0–14 in 1990 ( × 6); for 1999–2003 taken as 5/7 of that for 1999–2005 (Hoffmann *et al*, 2007).

b If the expected value for KR in 1984–1999 is (improbably) taken as twice that assumed, O=26, E=21.00, O/E 1.24, CI: 0.81, 1.82.
